# The dual blockade of the renin–angiotensin system in hemodialysis patients requires decreased dialysate sodium concentration

**DOI:** 10.1007/s11255-012-0320-z

**Published:** 2012-11-08

**Authors:** Rafał Zwiech, Agnieszka Bruzda-Zwiech

**Affiliations:** 1 Department of Kidney Transplantation, Dialysis Department, Norbert Barlicki Memorial Teaching Hospital No. 1, Medical University of Lodz, Kopcinskiego 22, 90-153 Lodz, Poland; 2 Department of Pediatric Dentistry, Medical University of Lodz, Pomorska 251, 92-213 Lodz, Poland

**Keywords:** Serum angiotensin-converting enzyme inhibitors, Angiotensin receptor blockers, Sodium concentration, Sodium gradient, Hemodialysis, Interdialytic weight gain, Thirst inventory

## Abstract

**Purpose:**

The study evaluated whether the dual blockade of the renin–angiotensin system may influence the sodium balance in hemodialysis.

**Methods:**

The study involved 148 hemodialysis patients (male 85, female 63), mean age 59.6 ± 12.9 years. Participants were randomly selected to receive either angiotensin-converting enzyme inhibitor (ACEI)—subgroup A—or dual blockade ACEI and angiotensin receptor blocker (ARB)—subgroup AA.

**Results:**

At baseline, in the A versus AA subgroups, the pre-dialysis sodium concentrations (mmol/l) were 137.7 ± 0.5 versus 137.9 ± 0.8, the sodium gradients 2.6 ± 0.5 versus 2.9 ± 0.4, interdialytic weight gain (IWG) (kg) 3.1 ± 0.2 versus 3.0 ± 0.3, and thirst inventory score (points) 18.1 ± 1.0 versus 19.0 ± 1.7, respectively. After 3 months of therapy, a decrease in sodium concentration to 134.5 ± 0.5 and the increase of its gradient to 5.5 ± 0.5 were noted in the AA subgroup. An elevation of mean interdialytic weight gain to 3.47 ± 0.2 and thirst score to 21.3 ± 2.1 was observed. No significant changes in subgroup A were found. One month of the dialysate sodium concentration being lowered from 140 mmol/l to 138 mmol/l was associated with reduced serum sodium concentration and gradient, decreased IWG and restored moderate thirst score in the AA subgroup (137.5 ± 0.6 and 2.9 ± 0.6, 3.0 ± 0.5 and 19.2 ± 1.3, respectively).

**Conclusions:**

The dual blockade of the renin–angiotensin system affects sodium balance, increasing the sodium gradient, thus elevating thirst sensation and enhancing interdialytic weight gain. In maintenance hemodialysis patients treated with both ACEI and ARB, lowered dialysate sodium levels should be prescribed.

## Introduction

Intermittent hemodialysis (HD) is the most commonly used renal replacement modality. During thrice-weekly sessions, the proper fluid volume balance must be restored. One way of doing this is by preserving the optimum sodium balance, which depends mainly on dietary salt intake and sodium removal during HD sessions [1]. With this in mind, increased dietary sodium ingestion is believed to be the main determinant of interdialytic weight gain in HD patients without hyperglycemia [2]. It leads to uncontrolled thirst, thus provoking fluid consumption and excessive weight gain [1, 3]. Chronic overhydration linked to sodium imbalance shows a number of cardiovascular manifestations and other complications such as chronic hemodilution and dilutional anemia, not to mention hypertension, while its reversal results in a decrease in morbidity and mortality [3–6].

The pathogenesis of thirst has not been clearly defined. Nevertheless, renin–angiotensin cascade activity, among other factors, may play a role by increasing sodium appetite [7]. Well-known antihypertensive drugs such as angiotensin-converting enzyme inhibitors (ACEI) and angiotensin receptor blockers (ARB) may reduce fluid intake and suppress drinking behavior in hemodialysis patients, though this has yet to be confirmed [8–10]. Although their thirst-reducing potential has been widely discussed, the mechanism of their action has not been investigated. Hence, we assume that sodium imbalance plays a key role in the treatment of excessive thirst and interdialytic excessive weight gain with ACEI and ARB.

The aim of the study was, therefore, to determine whether the dual blockade of renin–angiotensin system may influence the sodium balance in hemodialysis patients and subsequently affect thirst or interdialytic weight gain.

## Materials and methods

A prospective, randomized, open-label trial was conducted in 148 hemodialysis patients (male 85, female 63), mean age 59.6 ± 12.9 years. The mean time from starting hemodialysis was 13.5 ± 6.7 months. All subjects were recruited from the Dialysis Department of the Norbert Barlicki Memorial Teaching Hospital No. 1. The mean session time was 4 h and 15 min. The causes of end-stage renal disease included chronic glomerulonephritis in 36 patients, diabetic nephropathy in 55, adult polycystic kidney disease in 10, hypertension in 13, tubulointerstitial nephritis in 21, and unknown in 13 patients. The eligibility criteria for a patient to be included in the study were as follows: age between 18 and 80 years old, a fixed hemodialysis schedule of 3 times a week, and a stable clinical condition. The exclusion criteria comprised uncontrolled hypertension or recurrent symptomatic hypotension episodes, chronic heart failure (NYHA stage 4), severe acute infections requiring hospitalization and the administration of centrally acting sympathicolytics. All patients were advised to maintain their usual dietary habits.

Participants were divided into a study group—hypertensive patients (treated with combination of antihypertensive medication from 1 to 3 of type) and a control group—normotensive patients. Of the study group, two subgroups were formed by random selection of the hypertensive patients. The first (subgroup A) received a mean dose of 10 mg (single morning dose) of ACEI, that is, ramipril, while the second (subgroup AA) received a mean dose of 5 mg of ramipril in the morning and ARB, that is, losartan—a mean dose 50 mg—in the evening. These treatments allowed blood pressure below 140/90 mmHg before and 130/80 mmHg after hemodialysis to be achieved in all of the participants [11]. In both subgroups, antihypertensive treatment was not changed and doses were stable for the 4-month duration of the study.

The kidney replacement therapy was conducted on Fresenius 4008 dialysis machines exclusively. Standard bicarbonate dialysate fluid containing 140 mmol/l of sodium, 1.25 mmol/l of calcium, and 0.75 mmol/l of magnesium was used. The potassium concentration varied depending on the degree of the patient’s kalemia before the session. The dialysis adequacy was assessed with a single-pooled kT/V of average value 1.1–1.3. The dry weight was established based on clinical examination, blood pressure measurements, and whole-body composition spectroscopy [12]. The dialysis prescription did not change within 4 months of the study, except in the case of subgroup AA, in which the dialysate sodium concentration was reduced to 138 mmol/l after 3 months of ACEI and ARB therapy.

At baseline and after 3 and 4 months of the study on a second mid-week dialysis session, pre- and post-dialysis sodium concentration and sodium gradient were assessed. All measurements were carried out routinely in certified central hospital laboratory automatic analyzers. Interdialytic weight gain, defined as the difference between current body mass and dry weight (IWG), and blood pressure (BP) were measured before each hemodialysis, and the mean IWG and BP at every stage of the study were computed.

Additionally, all participants completed a survey evaluating thirst intensity. The dialysis thirst inventory is a questionnaire which consisted of 7 items, each with a 5-point Likert scale ranging from never (1) to always (5). The results ranged from a minimum 7 points (no thirst) to a maximum 35 points (enormous thirst). The thirst questionnaire was conducted together with the biochemical tests.

In all of participants, the mineral bone disorder associated with their renal anemia and kidney diseases was successfully treated according to the National Kidney Foundation Disease Outcomes Quality Initiative (NKF–KDOQI) recommendations [13, 14] as was diabetes mellitus [15]. The study and the control groups were age and sex matched, and significant parameters including mean hemoglobin, number of participants with preserved residual urination or diabetes, and mean session time were comparable. The results are summarized in Table 1.

**Table 1:** Structure and clinical characteristic of the study and the control group

	Study group		Control group
	Subgroup A	Subgroup AA	
*N*	47	47	54
Male (*n*)	28	29	28
Age (years)	60.1 ± 15.9	59.9 ± 11.5	58.5 ± 13.3
Diabetes (*n*)	18	19	22
HbA1c (%)	6.4 ± 0.6	6.6 ± 0.3	6.5 ± 0.4
Hemodialysis vintage (months)	11.1 ± 6.6	10.4 ± 5.3	10.1 ± 6.9
Dialysis session time (minutes)	250 ± 15	245 ± 20	255 ± 25
Kt/V	1.22 ± 0.12	1.21 ± 0.1	1.2 ± 0.08
Hgb (g/dl)	10.5 ± .1.2	10.9 ± 1.6	10.7 ± 1.3
Residual diuresis (*n*)	10	10	13
Volume (ml/day)	710 ± 110	690 ± 120	700 ± 140

HbA1c—glycosylated hemoglobin type A1c, Hgb—hemoglobin

Values are mean ± standard deviation (SD)

### Statistical analysis

The normality of distribution was checked by the Kolmogorov–Smirnov test. Comparisons within and between the study group and subgroups were performed using three-way ANOVA. The Fisher’s exact probability test was used for gender comparison. Differences were considered significant if *p* was <0.05. The results were expressed as mean ± standard deviation. Statistical analysis was performed using Statistica for Windows software (version 10.0).

We conducted our study in compliance with the principles of the Helsinki Declaration. The study protocol was approved by the Medical University of Lodz Bioethics Committee, Resolution Number RNN 147/09/KE. According to principles of good clinical practice (GCP), the informed consents have been obtained from all patients prior their inclusion in the study.

## Results

### Sodium kinetics

At baseline pre- and post-dialysis, sodium serum concentrations were similar in the subgroups and in the control group (*p* > 0.05 for all comparisons). After 3 months of treatment, pre-dialysis Na rapidly decreased in the AA subgroup. No changes in sodium concentration in other groups were observed. The correction of Na in dialysate resulted in restoring a balanced serum sodium concentration in the AA subgroup. All results are shown in Tables 2, 3, and 4.

**Table 2:** Parameters at baseline

	Study group		Control group
	Subgroup A	Subgroup AA	
Pre-dialysis Na (mmol/l)	137.7 ± 0.5	137.9 ± 0.8	137.7 ± 0.6
Post-dialysis Na (mmol/l)	138.3 ± 0.3	137.7 ± 0.6	138.2 ± 0.4
Pre-dialysis Na gradient	2.6 ± 0.5	2.9 ± 0.4	2.7 ± 0.6
Post-dialysis Na gradient	2.1 ± 0.5	2.0 ± 0.3	1.94 ± 0.5
Interdialytic weight gain (kg)	3.1 ± 0.2	3.0 ± 0.3	2.97 ± 0.4
Thirst score (pts)	18.1 ± 1.0	19.0 ± 1.7	18.6 ± 1.6
Systolic pressure (mmHg)	127 ± 12	128 ± 14	122 ± 9
Diasystolic pressure (mmHg)	78 ± 17	79 ± 12	76 ± 10

Values are mean ± standard deviation (SD)

**Table 3:** Parameters after 3 months of treatment

	Study group		Control group
	Subgroup A	Subgroup AA	
Pre-dialysis Na (mmol/l)	137.5 ± 0.4*	134.5 ± 0.5*’**	137.5 ± 0.7**
Post-dialysis Na (mmol/l)	138.0 ± 0.4*	136.5 ± 0.3*’**	138.1 ± 0.3**
Pre-dialysis Na gradient	2.46 ± 0.4*	5.5 ± 0.5*’**	2.3 ± 0.7**
Post-dialysis Na gradient	1.96 ± 0.4*	3.5 ± 0.2*’**	1.86 ± 0.3**
Interdialytic weight gain (kg)	2.85 ± 0.1*	3.47 ± 0.2*’**	2.91 ± 0.3**
Thirst score (pts)	17.6 ± 1.1*	21.3 ± 2.1*’**	18.4 ± 1.4**
Systolic pressure (mmHg)	125 ± 11*	130 ± 10*’**	124 ± 14**
Diasystolic pressure (mmHg)	75 ± 14	78 ± 13	76 ± 12

Values are mean ± standard deviation (SD)

ANOVA statistical significances with *p* < 0.01: *Subgroup AA versus A,**subgroup AA versus control group

**Table 4:** Parameters at the end of fourth month

	Study group		Control group
	Subgroup A	Subgroup AA	
Pre-dialysis Na (mmol/l)	137.2 ± 0.4	137.5 ± 0.6	137.4 ± 0.7
Post-dialysis Na (mmol/l)	138.1 ± 0.2	137.9 ± 0.5	138.1 ± 0.6
Pre-dialysis Na gradient	2.8 ± 0.7	2.9 ± 0.6	2.8 ± 0.8
Post-dialysis Na gradient	2.2 ± 0.5	2.1 ± 0.4	2.0 ± 0.3
Interdialytic weight gain (kg)	2.9 ± 0.2	3.0 ± 0.5	2.9 ± 0.4
Thirst score (pts)	19.1 ± 1.1	19.2 ± 1.3	18.8 ± 1.2
Systolic pressure (mmHg)	125 ± 13	127 ± 15	122 ± 10
Diasystolic pressure (mmHg)	76 ± 12	77 ± 13	75 ± 8

Values are mean ± standard deviation (SD)

### Sodium gradient

The initial pre- and post-dialysis sodium gradients were comparable in the study and control groups. Although a dual blockade of the renin–angiotensin system was seen to significantly increase pre- and post-dialysis sodium gradients in month 3 of treatment, the reduction of dialysate sodium decreased Na gradients in the AA subgroup. The results are collected in Tables 2, 3, and 4.

### Blood pressure, interdialytic weight gain, and thirst

At baseline, mean systolic and diasystolic blood pressure in hypertensive patients (subgroups A vs. AA) were similar. After 3 months of therapy with ACEI and ARB, blood pressure did not change, although systolic BP was significantly higher than in patients in subgroup A (*p* < 0.01). Although at the end of the study (month 4) blood pressure was comparable in both subgroups (*p* > 0.05) and systolic BP in subgroup, AA was significantly decreased in comparison with month 3 (*p* < 0.05). Twenty-six hypotension episodes, defined as a fall of BP below 90 mmHg or a decrease ≥20 mmHg from the pre-dialysis blood pressure, were observed in subgroup AA during the trial. In patients treated with a single blockade (ACEI), twenty-one hypotension episodes were noted. This difference was not found to be statistically significant.

The mean interdialytic weight in subgroup A did not differ at baseline in comparison with AA but after 3 months of therapy in patients with dual blockade, IWG was raised (*p* < 0.05) and was significantly higher in subgroup AA than in subgroup A (*p* < 0.01). At the end of the study, the mean IWG of the subjects in subgroup AA was comparable to that of subgroup A and to results noted in AA at baseline (all *p* > 0.05).

The initial mean thirst scores did not differ significantly between subgroups (*p* > 0.05); however, an assessment after 3 months of treatment indicated a significant increase in the AA subgroup (*p* < 0.05). In comparison with the control and subgroup A, the mean thirst score was significantly higher (*p* < 0.01). At the end of the treatment period, no differences between study groups and baseline results were observed (*p* > 0.05).

**Fig. 1:**
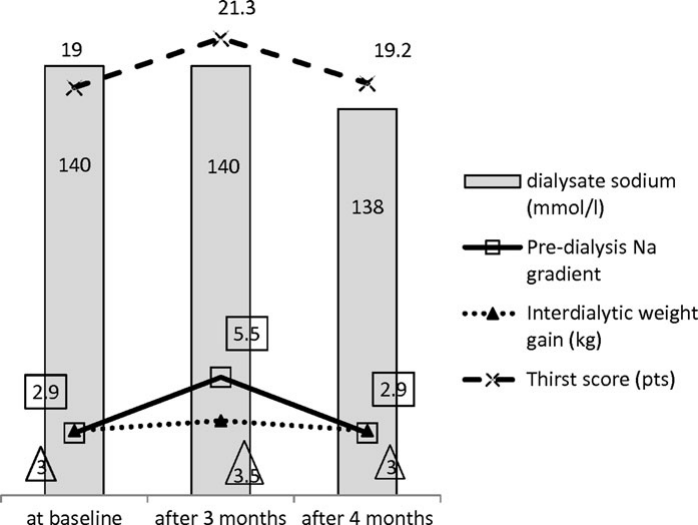
The changes in pre-dialysis serum sodium and sodium gradient, interdialytic weight gain and thirst sensation score in AA subgroup with the sodium dialysate prescription changes in the background

All results are presented in Tables 2, 3, and 4. Figure 1 shows the changes in pre-dialysis serum sodium and sodium gradient, interdialytic weight gain, and thirst sensation score in AA subgroup with the sodium dialysate prescription changes in the background.

## Discussion

Since excessive thirst and IWG were first defined as being strictly connected items which commonly occur in hemodialysis patients [16], various intervention strategies have been evaluated [8, 10, 17].

The influence of ACEI on IWG and thirst has been widely examined, and it was believed to play a positive role in the reduction of IWG [8, 10] although with some reservations [17]. Unfortunately, earlier studies had many limitations: mainly small numbers of participants or no wash-out period.

Also, some studies attempted to establish whether therapy with angiotensin receptor blockers (ARB) could reduce thirst and IWG [10]. Although the effectiveness of ACEI use in the reduction of thirst and IWG may be open for discussion, ARB seems to be ineffective [10, 18]. The only confirmed effect of ACEI and ARB administration was a reduction in angiotensin II and aldosterone level, as well as pseudo-normalization after drug withdrawal [8, 10]. Furthermore, studies on the administration of angiotensin receptor antagonists in hemodialysis patients are unsatisfactory due to their limited scope, in that they focus mainly on their safety [19].

Although the dual RAA blockade is not commonly administered, there are good theoretical reasons why combination therapy with ACEI inhibitors, and the use of ARB to block the binding of angiotensin II with its receptor, provides a more complete blockade of angiotensin effects. The ValHeFT study analyzed the benefits of the addition of valsartan to ACE inhibitor in 3034 chronic heart failure (CHF) patients [20]. It showed a significant decrease in the morbidity endpoints [20] and resulted in the reduction of cardiovascular death and hospitalization rates [20, 21]. Fluid overload and secondary CHF due to left ventricle hypertrophy are typical of maintaining hemodialysis patients, particularly in interdialysis periods, so that the introduction of a dual RAA blockade to those patients may be beneficial.

The decrease in renal salt and water excretion may lead to hypertension and, particularly, hypervolemia [1, 3] as well as thirst and fluid overload [22] in hemodialysis patients, while a reduction of dialysate sodium or dietary intake should, hence, improve the patient’s status. However, the important question is whether the reduction is beneficial to all dialysis patients, including those who are normotensive, those who are hypotensive, and those who lose renal salt [23]. Some authors indicate that no controlled studies of sufficient quality are available [24]. Therefore, it remains unclear what the most appropriate strategies for optimizing sodium corrections are and for whom they would be beneficial.

In our study, at baseline, the dialysate sodium concentration was set at 140 mmol/l, which generated a positive pre- and post-dialysis dialysate to the plasma sodium gradients which ranged from 2.6 in subgroup A to 2.9 in subgroup AA. Such a reduced Na gradient is associated with a significantly reduced thirst score and interdialytic weight gain [25, 26] and does not exacerbate intradialytic hypotension [27, 28].

After 3 months of treatment with a dual blockade of the renin–angiotensin system, pre- and post-dialysis sodium serum concentrations were lower than at baseline and decreased in subgroup AA in comparison with subgroup A and the control group. As a result, computed pre- and post-dialysis sodium gradients were higher. To maintain the osmolar set point, a positive dialysate to plasma Na gradient of over 3 mEq/l is associated with sodium retention and proportional fluid ingestion [26, 29]. Furthermore, the thirst score was increased up to a mean of 21.3 points, that is, from moderate to high, and elevated in comparison with the findings from subgroup A and the control group. Subsequently in the AA subgroup, an increase in mean IWG was observed, and despite a more efficient dual blockade of the renin–angiotensin system, elevated values for mean systolic blood pressure were noted.

A possible reason for these observations is that the introduction of ARB to the treatment impairs the sodium balance and switches the sodium set point, increasing its gradient and inducing post-dialysis thirst sensation and excessive fluid ingestion, thus leading to hypervolemia. It is worth noting that sodium removal during hemodialysis relies on both convective losses (78 %) and diffusive losses (22 %) [25]. In addition, increased IWG was found to result in sodium dilution and augmented ultrafiltration in the AA subgroup. Furthermore, enhanced IWG itself leads to a progressive increase of the sodium gradient.

Several studies have shown the ability of angiotensin-converting enzyme inhibitors to suppress thirst and effectively reduce interdialytic weight gain by modifying improper drinking behaviors in maintaining hemodialysis patients [8, 9]. In contrast, Masajtis et al. showed that adding an ARB to chronic ACEI therapy did not reduce the thirst sensation nor IWG in those patients [10].

Lack of effectiveness of ARB therapy in reducing thirst may play a pivotal role in both the increased IWG and lower pre-dialysis serum sodium concentrations noticed in our AA subgroup (those subjected to ACEI and ARB treatment). The double blockade of RAA has two further effects: not only does it increase plasma renin activity, but it also raises the angiotensin II serum concentration: Angiotensin II is synthesized by chymases (the angiotensin escape phenomenon) and cannot be bound to receptors already blocked by ARB. All of these factors exacerbate the thirst felt by hemodialysis patients [30, 31]. The consequent excessive water consumption by the patient may lead to electrolyte dilution and lower serum sodium concentration as a result.

Interestingly, a 1-month prescription of 138 mmol/l dialysate sodium allowed sodium balance normalization to be achieved, average interdialytic weight gain to be restored, thirst score to be reduced, and systolic blood pressure to be decreased. No further changes in antihypertensive treatment were needed.

However, there is still a need to explain the paradox of the “reversed” effect of a lowered sodium concentration in the dialysate: the greater the sodium gradient, the higher the IWG value [25, 26]. The reduction of sodium in the dialysate normalizes its gradient, which diminishes the sensation of thirst and excessive drinking related to the lowered serum sodium concentration due to serum dilution. An adjustment of dialysate sodium level seems to lead to stabilization of sodium levels in the serum, which would explain the nature of the decreased sodium gradient seen in subgroup AA, and its impact on the variables assessed in this study. This observation also confirms that a proper sodium gradient is of importance in helping chronic dialysis patients to maintain a correct sodium balance and prevent excessive fluid intake [32].

### Study limitations

The open-label design of the study. Also, the administration of numerous drugs may influence the thirst, IWG, and sodium balance [33]; for that reason, no changes in concomitant medications in any of subjects were made. The thirst assessment based on the dialysis thirst inventory was performed without an evaluation of xerostomia; however, we assumed that thirst is a subjective feeling and self-reporting methods seem to be the most appropriate.

## Conclusions

The dual blockade of the renin–angiotensin system affects sodium balance, increasing the sodium gradient, thus elevating thirst sensation and enhancing interdialytic weight gain. In maintenance hemodialysis patients treated with both ACEI and ARB, lowered dialysate sodium concentration should be prescribed.
